# The Key Digital Tool Features of Complex Telehealth Interventions Used for Type 2 Diabetes Self-Management and Monitoring With Health Professional Involvement: Scoping Review

**DOI:** 10.2196/46699

**Published:** 2024-03-13

**Authors:** Choumous Mannoubi, Dahlia Kairy, Karla Vanessa Menezes, Sophie Desroches, Geraldine Layani, Brigitte Vachon

**Affiliations:** 1 School of Rehabilitation Université de Montréal Montreal, QC Canada; 2 Centre interdisciplinaire en readaptation du Montreal Métropolitain Institut Universitaire sur la readaptation en déficience physique de Montreal Montréal, QC Canada; 3 Institute of Nutrition and Functional Foods Université Laval Quebec, QC Canada; 4 Centre nutrition, santé et société NUTRISS Université Laval Québec, QC Canada; 5 School of Nutrition Université Laval Québec, QC Canada; 6 Centre de recherche du centre hospitalier de l'universite de Montreal Montréal, QC Canada; 7 Département de médecine de famille et de médecine d’urgence Universté de Montréal Montreal, QC Canada; 8 Centre de recherche de l’Institut universitaire en santé mentale de Montréal Centre integre de sante et de services sociaux de l'Est-de-l'ile-de-Montreal Montréal, QC Canada

**Keywords:** telehealth, telemedicine, telenutrition, telemonitoring, electronic coaching, e-coaching, scoping review, type 2 diabetes, prediabetes, diabetes management, diabetes self-management, mobile phone

## Abstract

**Background:**

Therapeutic education and patient self-management are crucial in diabetes prevention and treatment. Improving diabetes self-management requires multidisciplinary team intervention, nutrition education that facilitates self-management, informed decision-making, and the organization and delivery of appropriate health care services. The emergence of telehealth services has provided the public with various tools for educating themselves and for evaluating, monitoring, and improving their health and nutrition-related behaviors. Combining health technologies with clinical expertise, social support, and health professional involvement could help persons living with diabetes improve their disease self-management skills and prevent its long-term consequences.

**Objective:**

This scoping review’s primary objective was to identify the key digital tool features of complex telehealth interventions used for type 2 diabetes or prediabetes self-management and monitoring with health professional involvement that help improve health outcomes. A secondary objective was to identify how these key features are developed and combined.

**Methods:**

A 5-step scoping review methodology was used to map relevant literature published between January 1, 2010 and March 31, 2022. Electronic searches were performed in the MEDLINE, CINAHL, and Embase databases. The searches were limited to scientific publications in English and French that either described the conceptual development of a complex telehealth intervention that combined self-management and monitoring with health professional involvement or evaluated its effects on the therapeutic management of patients with type 2 diabetes or prediabetes. Three reviewers independently identified the articles and extracted the data.

**Results:**

The results of 42 studies on complex telehealth interventions combining diabetes self-management and monitoring with the involvement of at least 1 health professional were synthesized. The health professionals participating in these studies were physicians, dietitians, nurses, and psychologists. The digital tools involved were smartphone apps or web-based interfaces that could be used with medical devices. We classified the features of these technologies into eight categories, depending on the intervention objective: (1) monitoring of glycemia levels, (2) physical activity monitoring, (3) medication monitoring, (4) diet monitoring, (5) therapeutic education, (6) health professional support, (7) other health data monitoring, and (8) health care management. The patient-logged data revealed behavior patterns that should be modified to improve health outcomes. These technologies, used with health professional involvement, patient self-management, and therapeutic education, translate into better control of glycemia levels and the adoption of healthier lifestyles. Likewise, they seem to improve monitoring by health professionals and foster multidisciplinary collaboration through data sharing and the development of more concise automatically generated reports.

**Conclusions:**

This scoping review synthesizes multiple studies that describe the development and evaluation of complex telehealth interventions used in combination with health professional support. It suggests that combining different digital tools that incorporate diabetes self-management and monitoring features with a health professional’s advice and interaction results in more effective interventions and outcomes.

## Introduction

### Diabetes and Nutrition

The prevalence of diabetes in Canada is constantly rising, and related health expenditures are among the highest in the world. In 2018, approximately 8% of the Canadian population was living with this disease, and it is predicted that in 2025, a total of 5 million people will be affected (ie, 12.1% of the population) [1,2]. According to estimates, type 2 diabetes accounts for 90% of all diabetes diagnoses in the general population, type 1 diabetes accounts for 9%, and other kinds of diabetes account for 1% [3]. The prevalence of diabetes has been closely linked to dietary and lifestyle factors prevalent within the country, such as high rates of obesity and sedentary behavior coupled with a diet often rich in processed foods. However, best practice guidelines suggest that the onset of type 2 diabetes can be delayed or prevented using early lifestyle change interventions. As prediabetes is characterized by elevated blood glucose levels that do not yet meet the diagnostic criteria for diabetes, the therapeutic management of diabetes and prediabetes is similar [4,5]. In both cases, a comprehensive approach is required to better control glycemia levels [6,7]. Many factors are involved in preventing the disease and achieving better disease control, such as changing lifestyles through education, supporting self-management, and preventing the development and progression of complications [8]. The Diabetes Canada clinical practice guidelines recommend that individuals with diabetes receive personalized nutrition counseling by a registered dietitian to optimize glycemic control and weight management [3]. Strategies include caloric reduction for individuals who are overweight; the incorporation of low glycemic index carbohydrates; and the adoption of a Mediterranean, Nordic, Dietary Approaches to Stop Hypertension (DASH), or vegetarian diet because they are rich in protective foods [3]. These interventions are supported by evidence demonstrating improvements in glycated hemoglobin (HbA_1c_) levels, metabolic outcomes, and reductions in hospitalization rates. As stated in the Diabetes Canada clinical practice guidelines, the care offered should be organized around the needs of people with diabetes (and of their families and close friends) because patients must be active participants for optimal engagement in self-managing their condition [4,8]. This active patient participation must be facilitated by a multidisciplinary team (nurses, dietitians, and physicians) that offers education and self-management support. Changing dietary behaviors poses a considerable challenge for people living with diabetes, yet it is a vital means of preventing the associated complications [4]. Monitoring with a dietitian’s involvement has proven effective in supporting such behavior changes [4]. Again according to the Diabetes Canada clinical practice guidelines, all people living with diabetes should receive the services of a dietitian [4]. It has been shown that diet monitoring with a dietitian’s involvement can alone reduce HbA_1c_ levels by 1% to 2% [4]. In addition, recent evidence underscores the advantages of using telehealth to foster adherence to medical recommendations and self-management [4,5,9]. Scientific literature has shown the benefits of telehealth in Canada for diabetes management [3,10]. These technological innovations facilitate patient monitoring and promote the use of different interventions that can support lifestyle changes through, for example, remote support, the telemonitoring of glycemia levels, reminders about taking medication, and the use of a food diary. These innovations also allow this information to be shared with the health care team. In 2018, the Diabetes Canada clinical practice guidelines advocated for the use of telehealth in disease management programs to improve self-management in underserved communities and to facilitate consultation with specialized teams, highlighting its effectiveness and the importance of integrating it into shared care models [3].

### Telehealth and Diabetes Self-Management

Telehealth refers to “the use of communications and information technology to deliver health and health care services and information over large and small distances” [11]*.* In the same field of application, telemedicine refers to the exchange of medical information using information and communication technologies to improve a patient’s health condition and is delivered by at least 1 health professional [12]. Telemedicine services are provided using various means, including the telephone, internet, email, mobile apps, SMS text messaging, photographs, and videos. New technologies are revolutionizing the health care field by creating new prospects for various care delivery modalities [13]. They are thus paving the way for innovations and represent a real benefit in the face of new health care challenges, such as the aging population, rising health care costs, and the unprecedented challenges posed by pandemics such as the COVID-19 pandemic [6]. Particularly in Canada, the public health care system faces challenges often associated with overcrowded clinics, long wait times, and limited resources [7]. Through remote consultations and continuous monitoring, telehealth has the potential to relieve pressure on health care facilities, improving resource allocation and optimizing patient flow management in the public health care system. As such, telehealth would be a pertinent response to public health organizational challenges in the Canadian context, where the universal health care system aims to provide equitable and accessible care to all residents.

The day-to-day management of type 2 diabetes can be a complex challenge. Patients must monitor their blood glucose levels regularly, take medication on a precise schedule, adopt a balanced diet, and maintain adequate physical activity [7]. However, these requirements can be difficult to meet owing to time constraints, a lack of knowledge, or limited resources. In addition, fluctuations in blood glucose levels can occur unpredictably, increasing the risk of short- and long-term complications [7]. In particular, nutrition plays a fundamental role in diabetes management. Dietary monitoring, nutrition education, and the personalization of dietary recommendations are key aspects in optimizing health outcomes for patients with diabetes. Using digital technologies, it is possible to offer ongoing personalized nutrition support, enabling patients to make informed dietary decisions and maintain adequate glycemic control.

Recent evidence points to the enormous potential of using health technologies to facilitate access to care, patient adherence to their treatment plan, and self-management [14]. Many experts point out that diabetes is a chronic disease best adapted to self-management through telehealth [14-19]. Technological innovations have been developed to support lifestyle changes and facilitate patient monitoring. Telehealth offers a range of potential benefits for people with type 2 diabetes. Continuous monitoring of blood glucose levels using connected sensors enables patients to receive real-time information on their blood glucose levels and be alerted to abnormal variations [2,3]. This enables them to take immediate action to correct blood glucose levels and avoid complications. In addition, telehealth facilitates access to specialized care by enabling patients to consult health professionals remotely. This reduces geographic barriers and enables patients to receive personalized advice, education, and support tailored to their specific needs [9]. Regular monitoring and feedback as well as the use of digital tools encourage patients to better understand their condition, make informed decisions, and improve their quality of life [8]. According to recent systematic reviews and meta-analyses, these telehealth interventions involving everyday web-based and mobile technologies help reduce HbA_1c_ levels, allow for better daily glycemic control, promote an increase in physical activity, and improve dietary habits [20,21]. Connected blood glucose meters enable more convenient and accurate monitoring of blood glucose levels, whereas web-based platforms offer a web-based space for education, support, and communication with health professionals [14,15]. Teleconsultation enables patients to consult their physicians and specialists remotely, reducing travel and time constraints [15,16].

Combining self-management technologies with clinical expertise, social support, and health professional involvement can allow the development of telehealth solutions better adapted to the therapeutic management of patients with a chronic disease. Telehealth interventions using this combination are therefore expanding [22], but they present both advantages and limitations [12]. Telehealth enables improved care coordination, personalized interventions, and tailored patient education. However, it can lead to an increased workload for health care providers and raise data privacy concerns. The tension between interventions focused on service delivery and those involving health care providers highlights the importance of striking a balance between patient autonomy and medical expertise. An integrated collaborative approach involving both patients and health care providers may offer the best digital health outcomes. However, further studies are needed to fill the gaps in the literature, focusing on comparative studies with usual care, the evaluation of adherence, and long-term accessibility to optimize the use of telehealth in the self-management of type 2 diabetes.

To the best of our knowledge, no literature review has been conducted to identify the key digital tool features of such interventions. Nonetheless, improving knowledge on this subject could advance the development of more effective telehealth interventions for people with diabetes.

The primary objective of this scoping review was to identify the key digital tool features of complex telehealth interventions used for diabetes self-management and monitoring with health professional involvement that help improve health outcomes. The secondary objective was to identify how these key features should be developed and combined to optimize their contribution to improving health outcomes. Although our review draws from global scientific literature, the intent is to inform the future development of telehealth technologies, with a particular emphasis on the Canadian health care context. This focus stems from the recognition that although universal principles may guide the development of digital health tools, the specific features and their implementation must be tailored to meet the unique needs, regulations, and health care infrastructure of Canada. Our review aims to explicitly identify the characteristics of digital tools that have been shown to be effective in improving patient engagement, improving self-management, and leading to better health outcomes in diabetes care. By systematically cataloging these characteristics, we can provide a model for the design, development, and implementation of future telehealth interventions, provided we keep in mind specific requirements of the Canadian health care context, such as compliance with telehealth policies, local health care, patient privacy laws, and existing health IT infrastructure. In this study, *improving health outcomes* encompasses both the positive effects of the intervention on behavior changes (eg, eating healthier foods or performing physical activity) and the positive impacts on the health condition (eg, improved blood glucose levels or blood pressure).

## Methods

### Overview

Scoping reviews exhaustively synthesize the evidence to map a vast, complex, or emerging field of study and identify gaps in the literature, ultimately highlighting priorities for future studies in the field [23]. We chose this method because telehealth has emerged in different formats and offers solutions to various pathologies. We structured our scoping review according to the five steps developed by Arksey and O’Malley [24] and the revisions made by Levac et al [25]: (1) identifying the research question; (2) identifying relevant studies; (3) selecting the studies; (4) charting the data; and (5) collating, summarizing, and reporting the results. The procedure, which is described in the following subsections, was conducted in accordance with the PRISMA-ScR (Preferred Reporting Items for Systematic Reviews and Meta-Analyses Extension for Scoping Reviews) checklist (Multimedia Appendix 1) to ensure rigorous and transparent reporting of the methodology and findings [26]. Several additional recommendations made by Levac et al [25] were also followed: clearly articulate the research question for the scoping review, have 2 researchers independently review the full articles to determine their inclusion, have the research team collectively develop the data-charting form, and continually extract data.

### Identifying the Research Questions

This review seeks to answer the following research questions:

1. What are the key digital tool features of complex telehealth interventions used for diabetes self-management and monitoring with health professional involvement that help improve health outcomes?
2. How should these key features be developed and combined to help improve health outcomes?

These questions stem from the lack of consensus in scientific literature on the conceptual development, implementation, and evaluation of telehealth solutions. The research questions and objectives were developed based on the research team’s expertise and a preliminary analysis of the literature on the subject. In accordance with scoping review methodology, this review included studies that used different approaches and research designs.

In this review, we applied the World Health Organization definition of telemedicine: “The delivery of health care services, where distance is a critical factor, by all health professionals using information and communication technologies for the exchange of valid information for diagnosis, treatment and prevention of disease and injuries, research and evaluation, and for the continuing education of health care providers, all in the interests of advancing the health of individuals and their communities.” Furthermore, in the context of telehealth technology, the term *features* refers to the various components or tools that enable the various activities associated with remote health care delivery.

### Identifying and Selecting the Studies

The search strategy was developed in collaboration with a Université de Montréal librarian specializing in health. The keywords based on *telehealth*, *nutrition*, and *diabetes* were identified by examining relevant articles, their references, and the associated keywords (Multimedia Appendix 2). A systematic search was performed in the MEDLINE, CINAHL, and Embase databases, covering the period from January1, 2010, to March 31, 2022. Our search efforts were focused on these databases because they are repositories where studies related to health and nutrition can be found. Only articles published since January 1, 2010, were selected to account for the widespread adoption of smartphones. By extending our review to cover more than a decade, we were able to capture the significant developments in mobile apps and smartphone use, which are pivotal in digital health. We also perused the bibliographies of the included articles to identify any additional studies. Only articles published in peer-reviewed scientific journals were examined. As proposed by the framework developed by Arksey and O’Malley [24], a quality assessment was not performed because it is not deemed essential for exploratory studies. The methodological rigor of the published articles was not an inclusion or exclusion criterion; instead, the articles were examined to substantiate the results and the discussion.

Given the rapid development of new technologies, only articles on complex telehealth interventions for managing diabetes published in the 12 years covering the period from January 1, 2010, to March 31, 2022, were retained. We used an iterative process to develop the inclusion and exclusion criteria during our searches to ensure a selection of studies more closely aligned with the research question. The searches were limited to scientific publications in English and French that either described the conceptual development of a complex telehealth intervention combining self-management and monitoring with health professional involvement or evaluated its effects on the therapeutic management of patients with type 2 diabetes or prediabetes. For inclusion in this review, the complex interventions had to be digital, have a patient interface, and concern type 2 diabetes or prediabetes self-management or monitoring. We excluded studies (1) not using a nutritional approach to investigate telehealth interventions, (2) involving a single component, (3) not integrating at least 1 health professional, (4) concerning type 1 diabetes or gestational diabetes, (5) involving populations aged <18 years, and (6) lacking empirical data (eg, literature reviews). All search results were imported into the Covidence reference management software (Veritas Health Innovation Ltd), and duplicates were removed [27].

The review team comprised CM, DG, KVM, and BV. These 4 researchers determined the inclusion of relevant studies based on the title and abstract; CM and BV determined the selection based on the full-text articles. Differences were discussed in detail until a consensus was reached. The full texts of the relevant articles were retrieved for more in-depth analysis (CM).

### Charting the Data

The research team developed a data extraction table. It included the following information: study characteristics (eg, title, participants, the results of interest, and effectiveness), intervention characteristics (eg, a brief description of the intervention, the components of self-management, and the components of monitoring with health professional involvement), and the benefits and limitations of both the intervention and the study according to the authors or reviewers.

### Collecting, Summarizing, and Reporting the Results

Again according to the framework developed by Arksey and O’Malley [24] and the revisions by Levac et al [25], descriptive web-based abstracts and thematic analyses performed with NVivo software (release 1.7; Lumivero) were used for data analysis, yielding an approach resembling that of a narrative review. In conducting our thematic analysis, we adopted a qualitative approach to discern the impact of telehealth interventions with health professionals on the health outcomes of patients with diabetes. Through meticulous data immersion and iterative coding, we identified recurring patterns that we then shaped into themes. An initial list of these codes, forming a codebook, was iteratively refined during the data analysis process [28]. Once the codes were established, it enabled a comprehensive review of their interrelationships, aiding in the identification of the key digital tool features of complex telehealth interventions used for diabetes self-management and monitoring with health professional involvement that help improve health outcomes. These themes were refined against the data set to ensure coherence and direct relation to our research objectives. By integrating concrete examples from the data, we were able to provide a rich, detailed description of the telehealth features, thereby adding depth to our findings and ensuring that they were both representative of real-world practices and aligned with our research questions.

## Results

### Overview

**Figure 1:**
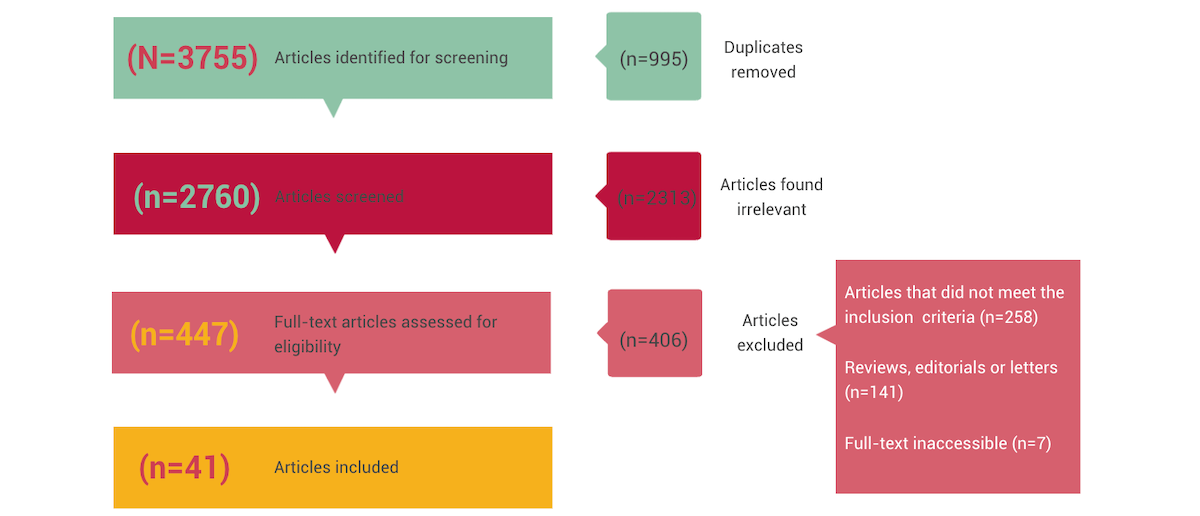
Flow diagram of study selection.

The database searches identified 3755 articles, from which 995 (26.5%) duplicates were removed. The 2760 remaining articles underwent an initial screening based on the abstract and title, after which 2313 (83.8%) were excluded. The full-text screening involved assessing 447 articles, of which 406 (90.8%) were deemed ineligible because the studies did not meet the inclusion criteria (n=258, 63.7%); were literature reviews, editorials, or letters (n=141, 34.8%); or the full texts were inaccessible (n=7, 1.7%; Figure 1). Thus, of the 3755 articles identified from the database searches, 42 (1.12%) were ultimately included in this scoping review (Multimedia Appendix 3 [29-70]). The qualitative analysis of the 42 articles using NVivo (release 1.7) yielded the coding of 1520 references, divided among 113 codes.

### Characteristics of the Studies

**Figure 2:**
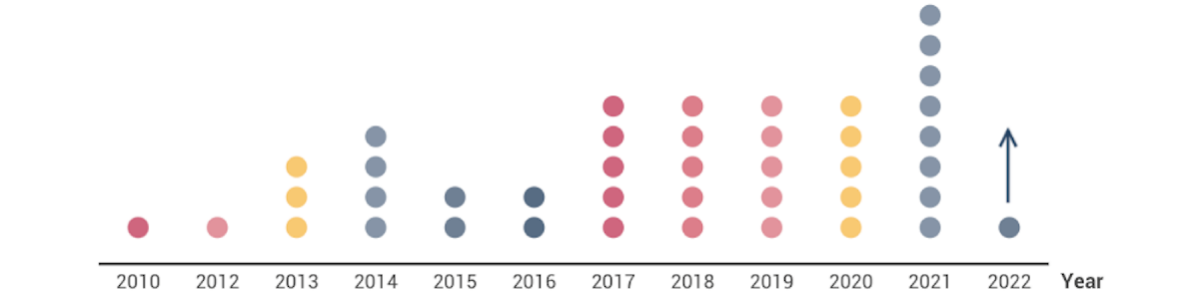
Years in which the studies were published. Each circle represents 1 study.

The 42 studies were published between January 1, 2010, and March 31, 2022, with as many as 28 (67%) published within the past 6 years [29-56]. We found that, in 2021, nearly twice as many articles were published on the topic as in each of the previous 4 years (Figure 2).

**Figure 3:**
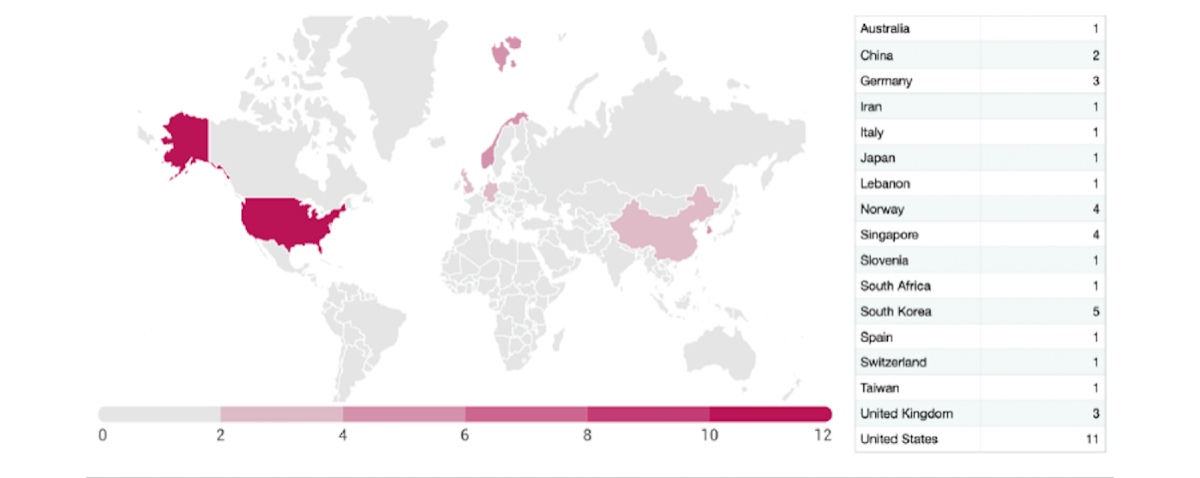
Countries in which the studies were published. The dots represent articles and the x-axis denotes the years.

Information on complex telehealth interventions used for diabetes self-management and monitoring with health professional involvement was obtained for 18 countries. Of the 42 studies, 11 (26%) were conducted in the United States [30,31,39,48-51,57-59]; 5 (12%) in South Korea [37,41,44,60,61]; 4 (10%) in Singapore [29,43,46,55]; 4 (10%) in Norway [32,62,63]; 3 (7%) in the United Kingdom [33,35,38]; 3 (7%) in Germany [40,45,56]; 2 (5%) in China [47,64]; and 1 (2%) each in Australia [54], South Africa [65], Spain [66], Iran [52], Italy [67], Japan [42], Lebanon [34], Slovenia [36], Switzerland, and Taiwan [68] (Figure 3).

### General Characteristics of the Intervention

**Figure 4:**
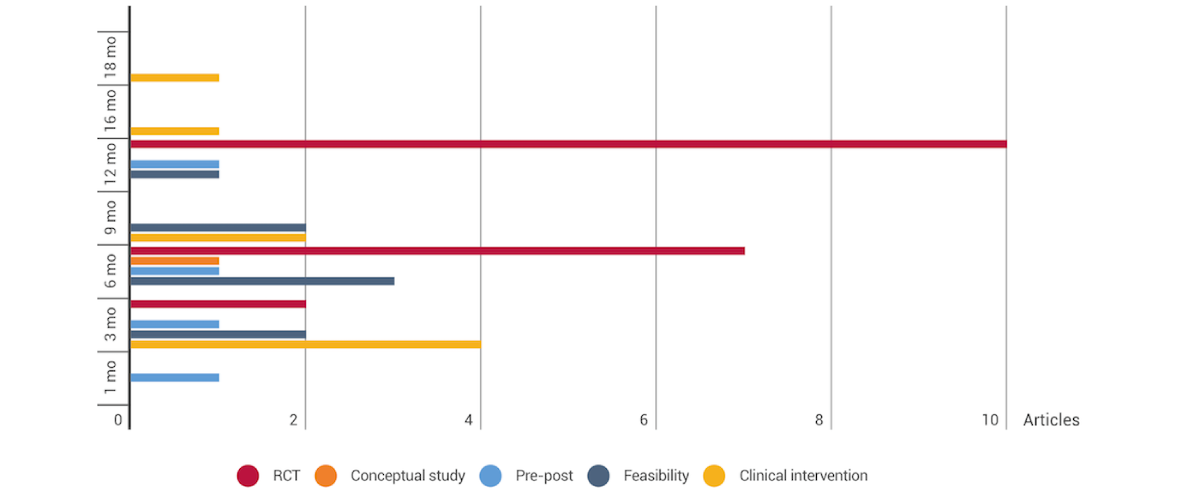
Durations of the interventions. RCT: randomized controlled trial.

One-third (14/42, 33%) of the studies were randomized controlled trials [35,36,40-42,46,47,55,57-59,62,64,67], with most of them (n=12, 86%) ranging from 6 months to 1 year in duration. Of the 42 studies, 9 (21%) were feasibility studies, with the interventions ranging from 3 months to 1 year in duration [33,38,39,43,44,51,53,56,71]; 8 (19%) were interventional studies, with the interventions ranging from 3 to 18 months in duration [30,31,34,48,50,54,63,68]; 5 (12%) were conceptual studies lasting 6 months [45,49,52,65,66]; and 4 (10%) were pre-post studies, in which the interventions ranged from 1 month to 1 year in duration [29,37,60,61] (Figure 4).

### Health Professional Involvement

Of the 42 studies, 21 (50%) included physicians [29-32,36,40-42,45,47-51,53,58,60,64,66,68,69], 16 (38%) involved dietitians [29,31,33,35,39,43,46,54,56,59,60,65,68-70, 72], 12 (29%) involved nurses [31,32,36,41,55,58-62,68,69], 4 (10%) involved psychologists [31,33,67,69], 4 (10%) involved physical educators [29,33,35,60], and 3 (7%) involved case managers [36,60,68]. Finally, of the 42 studies, 13 (31%) involved a multidisciplinary team [29,31-33,35,36,41,58-60, 68,69,72], and 22 (52%) involved only 1 clinician [30,39,40,42,43,45-51,53-56,61,62,64-66,70] (Multimedia Appendix 4 [29-70]).

### Characteristics of Digital Self-Management

The interventions under study involved the use of a mobile app [29-34,36-39,41-46,49,51-53,55,59-65,67,69,70] or a web portal [32,35,36,40-42,44,45,47,48,52,57,58,60,63,66-68,70], usually coupled with a blood glucose meter to optimize diabetes self-management [37,38,40,41,43,44,46,48-51,53,55,57,59-64, 68,69]. Other Bluetooth-connected devices were used in some of the interventions (10/42, 24%), such as a Bluetooth-connected weight scale [31,40,42,43,46,48], a pedometer [40,43,45], an accelerometer [33,42], a Bluetooth-connected smartwatch [49], and a tensiometer [42].

The types of data collected concerned the monitoring of glycemia levels through, for example, the visualization of a blood sugar curve over time [29,32,37,38,41,43-49,51-53,55, 59-66,68,69]; physical activity monitoring using, for example, a pedometer [33,35,40,43,45,46,56,61,67]; diet monitoring using, for example, a food diary [29-33,35,37,39,41,43-45,47, 49,52,53,55,56,59,61-63,67-69]; medication monitoring through, for example, adherence monitoring or the possibility of issuing remote prescriptions [30,50]; and other health data monitoring (weight, BMI, and laboratory tests) [29,32,33,40,43,45-47,53,56,60,65,66]. Other features made it possible to ensure continuity of care by, for example, generating reports [34,38,42,45,47,52,60,66,67,70]; supporting therapeutic patient education; and ensuring support from a health professional to help patients learn and develop skills to independently manage their chronic disease and improve their quality of life [15,16].

On the basis of our analysis of the literature, we classified the key digital tool features that can have a positive impact on intervention outcomes into eight categories: (1) monitoring of glycemia levels, (2) diet monitoring, (3) physical activity monitoring, (4) medication monitoring, (5) therapeutic education, (6) health professional support, (7) other health data monitoring, and (8) health care management (Multimedia Appendix 5 [29-70]).

### Key Digital Tool Features With Positive Impacts on the Health Condition

#### Monitoring of Glycemia Levels

Of the 42 studies, 22 (52%) incorporated a blood glucose meter to precisely monitor blood glucose levels during interventions; the blood glucose meter allowed the visual tracking of blood sugar curves by the patient and health professionals [37,38,40,41,43,44,46,48-51,53,55,57,59-64,68,69]. In addition, 3 (7%) of the 42 studies included blood glucose meters permitting real-time continuous blood glucose monitoring [29,30,50].

Of the 42 studies, 4 (10%) included an alert system [36,52,68,73]: “The online diabetes self-management system sent an SMS text message to care providers when the data exceeded the alerting range” [68]; “The application automatically sent users reminders by simple e-mail and SMS: ‘Please enter your blood sugar/or other parameters into the eDiabetes application’” [36]. Of the 42 studies, 9 (21%) included a bolus dosing system [32,38,45,55,57-59,66,74]: “An optional bolus dosing feature was available as an algorithm on the e-diary that allowed the patient to generate a premeal bolus insulin dose” [57]. Of the 42 studies, 2 (5%) allowed the remote prescription of real-time continuous blood glucose monitoring devices [30,50].

The 42 studies used different indicators to collect glycemic control data, such as (1) HbA_1c_ levels in 27 (64%) studies, monitored through blood tests [29-34,36,40,41,43,44,46-48, 51,54,55,57-60,62-64,67-69]; (2) blood glucose levels in 24 (57%) studies, monitored using data recorded by a blood glucose meter or a blood test [32-34,36,40,41,43,44,46-48, 50,53-55,57-60,63,64,66,68,69]; and (3) hypoglycemia events in 4 (10%) studies [55,57,60,69], based on self-reports or alert systems after the recording of blood glucose levels with a blood glucose meter. All interventional studies included in the review reported a reduction of between 0.433 mmol/L and 1.554 mmol/L in fasting blood glucose levels. The studies reported a statistically significant decrease in HbA_1c_ levels ranging from 0.5% to 1.65% [34,40,41,57,68], as well as a drop of up to 1.554 mmol/L in blood glucose levels [29-31,36,41-44,46,48,56, 57,59,61,62,68,69].

#### Diet Monitoring

Of the 42 studies, 13 (31%) included a meal planning system, with features such as generating shopping lists and recipes and calculating caloric intake [29,31,35,38,43,44,46,48,49, 52,53,65,68]; and 27 (64%) included a food diary system that could be shared with the health professional for comment [29-33,35,37-39,41,43-47,49,52,53,55,56,59,61-63,67-69]. Patients logged their data using a list of foods or by taking photographs. A caloric intake–counting feature was available in 11 (26%) of the 42 studies [29,35,38,43,44,48,49, 52,53,65,68]. Of the 42 studies, 5 (12%) included a carbohydrate-counting system [32,46,49,53,66]: “The app provided an automated individualized calorie limit which was computed based on body weight, gender, age and activity level. The total daily carbohydrate intake was restricted to 40% of total daily calories” [46]; “From the nutrition screen, the test persons manually entered carbohydrate values for their meals or scanned products to import the carbohydrate data into the app” [53]. Of the 42 studies, 18 (43%) included pedagogical material, particularly nutrition education and knowledge evaluation [31,33-37,46-48,51,52,55,59,60,63,64,66,69]. To collect data on diet, the studies used the data logged on mobile or internet platforms or obtained from food diaries, 24-hour reminders, or calorie counting [32,46,49,53,66]. The health professionals evaluated diet quality using the shared data or validated questionnaires (eg, the Healthy Eating Index). The studies reported a better understanding of nutritional issues, greater confidence in maintaining a healthy diet, and an improvement in dietary behavior [30,31,40,41,44,61,68,70].

#### Physical Activity Monitoring

Of the 42 studies, 6 (14%) monitored physical activity using a Bluetooth-connected device (Bluetooth-connected watch [49], pedometer [40,43,45], or accelerometer [33,42]), 8 (19%) used step counting via a Bluetooth-connected pedometer or a smartphone-integrated feature [33,35,40,45,46,56,61,67], and 16 (38%) included a graphic monitoring tool for monitoring physical activity [29,32,35,37,41,43-45,49,52,55,62-65,69]. These graphs were generated automatically using pedometer data or after patients’ manual logging of their activities based on a list of predefined physical activities. A caloric expenditure–counting feature was often available: “Type, time, and intensity of any completed physical activity, which could be translated into calories burned. (BCT: prompt self-monitoring of behavior; provide feedback on performance)” [35]. The studies used data logged on mobile or internet platforms and obtained from pedometers, accelerometers, or self-reported physical activity diaries to collect physical activity data. These data made it possible to adjust the automated recommendation messages and the messages from the health professionals with whom the data were shared. The studies reported a trend toward increased weekly physical activity owing to the technology-motivated engagement (eg, Chen et al [68] report a significant increase in physical activity; *P*<.001) [30-38,41-47,50,54,55,57,60,62-64,66-69].

#### Medication Monitoring

Of the 42 studies, 16 (38%) included a medication adherence–tracking device [30,32,37,38,41,45,49,52,53,55,59, 61,63-65,68], half of which (n=8, 50%) had a reminder feature [32,37,45,52,55,61,63,68]. Of the 42 studies, 6 (14%) included an insulin dose–adjustment device used by the health professional or patient (eg, using a bolus dose algorithm) [29,40,48,57,66,69]. Regarding the medication data collected, of the 42 studies, 6 (14%) reported medication adjustments [29,40,48,57,66,69], 7 (17%) analyzed the monitoring of prescribed insulin doses [30,32,55,57,58,66,68], and 5 (12%) administered questionnaires on medication adherence [31,34,50,57,67]. Finally, 4 (10%) of the 42 studies reported decreased oral antidiabetic doses after the interventions [31,40,48,68].

#### Therapeutic Education

Patients were provided various pedagogical tools to support their therapeutic education in 20 (48%) of the 42 studies [31,33-37,43,46-48,51,52,55,59,60,63,64,66,69,70]. Among these 20 studies, web-based course modules were used in 4 (20%) [43,48,63,66]. Other tools were used to advance nutritional literacy [31,35,46,59]; or the tools talked about or referred to relevant articles on topics such as using a blood glucose meter, diabetes complications, physical activity, and tobacco use [33,35,36,43,46,48,52,55,59,66,69]. Finally, 2 (10%) of the 20 studies proposed meditation or mindfulness exercises [51,55]. Personalized recommendation tools were used in 11 (26%) of the 42 studies [29,37,45-48,51,52,60,63,66]. These recommendations were either delivered by a health professional after an analysis of the patient’s logged data, generated automatically by an artificial intelligence algorithm, or planned according to a therapeutic education protocol. The pedagogical materials were often supported by electronic notebook tools where patients could jot down topics to discuss with their health professionals [52,64,67].

#### Health Professional Involvement

Among the 42 studies, communication between the health professional and patient was ensured through a chat feature in 13 (31%) studies [31,35,43,46,47,49,51,52,59,60,63,66,68], by email in 7 (17%) studies [31,33,36,43,66,67,71], by SMS text messaging in 14 (33%) studies [29,36,37,41,42,44,48,54,58, 62,63,67-69], by telephone calls in 13 (31%) studies [31,33,40,48,55-58,60,62,67-69], and by videoconferencing in 4 (10%) studies [55,60,67,68].

Of the 42 studies, 33 (79%) included a tool for displaying patient data [30,32-37,39,41-49,51-59,63-66,68-70], one-third of them (n=11, 33%) in real time, in the form of a graphic report. Of the 42 studies, 3 (7%) included a decision support tool [34,45,64], whereas 12 (29%) included a tool for setting and monitoring therapeutic goals that could be shared by the care provider and patient [29,32,33,37,45,46,53,59,61,63,68,69].

#### Other Health Data Monitoring

The monitoring of other health data concerned weight loss. Of the 42 studies, 16 (38%) monitored weight using a graphic representation over time [29,31-33,40,42,43,45-48, 53,56,60,65,66]. Of these 16 studies, 6 (38%) collected automated data using a Bluetooth-connected weight scale [31,40,42,43,46,48]. In addition, 7 (17%) of the 42 studies enabled the sharing of blood test results [38,40,60,61,64,65,68]. Kobayashi et al [42] used a Bluetooth-connected tensiometer to transmit blood pressure readings to a cloud-based server, making it possible to summarize and present the data to patients and their primary care physicians to promote self-management, monitoring, and follow-up. The studies reported a statistically significant reduction in weight ranging from 3 to 6.2 kg [29,40,43,46,56,60] and in BMI ranging from 1.6 kg/m^2^ to 4 kg/m^2^ [29,34,42,48,56,60].

#### Health Care Management

Of the 42 studies, 20 (48%) included personal spaces in their technologies [31-35,38,40,42,45,47,49,51-53,61,63,66-68,70]. In these spaces, it was possible to view a dashboard summarizing the logged health data, monitor exchanges with health professionals, and generate reports that could be shared by the patient and downloaded by the health professionals for inclusion in the medical file [34,38,42,45,47,52,60,66,67,70]. Social support was promoted through links to social networks in 6 (14%) of the 42 studies [31,35,37,41,44,48]. Of the 42 studies, 6 (14%) included a web-based appointment scheduling tool, facilitating monitoring and follow-up by the health professionals [32,33,35,53,64,67]. Finally, Holmen et al [69] made technical support available 7 days a week to users of their technology.

#### Combination of Interventions

Studies showing significant positive results were those combining the involvement of a health professional with the monitoring of glycemia levels, diet, physical activity, and medication [41,57,61]. Of the 42 studies, 1 (2%) combined support from a health professional with the monitoring of glycemia levels, diet, and physical activity; therapeutic education; and a follow-up of body weight [29]. Some of the studies (7/42, 17%) only added to the involvement of a health professional the monitoring of glycemia levels and physical activity (n=1, 14%) [40], the monitoring of glycemia levels alone (n=2, 29%) [51,58], diet and medication monitoring with therapeutic education (n=1, 14%) [31] or without therapeutic education (n=1, 14%) [35], diet monitoring and therapeutic education (n=1, 14%) [70], and physical activity and body weight monitoring (n=1, 14%) [42]. Of the 42 studies, 2 (5%) with positive significant results evaluated the combination of a health professional and the monitoring of glycemia levels, diet, and medication (n=1, 50%) [30] and therapeutic education and body weight follow-up (n=1, 50%) [34]. Most often (23/42, 55%), the combined strategies involved a health professional and the monitoring of glycemia levels and diet (Multimedia Appendix 6 [29-31,34,35,40-42,51,57,58,61,70]).

## Discussion

### Principal Findings

**Figure 5:**
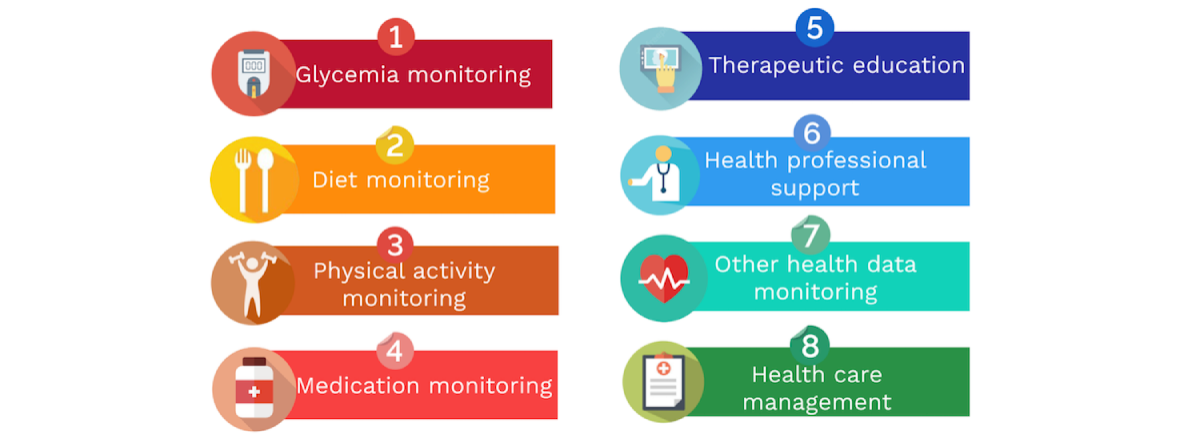
Classification of digital features for diabetes self-management and monitoring.

This study mapped telehealth interventions tailored to the needs of patients with type 2 diabetes supported by a health professional. This review—despite the range of scientific literature available; the complex nature of these interventions; and the heterogeneity of study designs, populations, organizational care contexts, measures, and result indicators used—revealed a trend suggesting the effectiveness of telehealth interventions with health professional involvement in improving health outcomes. The use of everyday technologies in these interventions could facilitate their accessibility and usability, which would facilitate their implementation in the longer term. On the basis of our exploration of the literature, we were able to classify the key features of digital tools that may have a positive effect on intervention outcomes into eight categories: (1) monitoring of glycemia levels, (2) diet monitoring, (3) physical activity monitoring, (4) medication monitoring, (5) therapeutic education, (6) health professional support, (7) other health data monitoring, and (8) health care management (Figure 5).

The duration of the interventions varied significantly among the studies, with interventions lasting 1 month to 18 months. A recent meta-analysis on the effectiveness of telemedicine application for chronic diseases found that for people living with type 2 diabetes, HbA_1c_ levels began to decrease after up to 12 months of telemedicine intervention compared with interventions lasting 6 months [75]. These results were also supported in a study by Timpel et al [76], where HbA_1c_ levels began to decrease in participants after 12 months of long-term telemedicine intervention. Given that the HbA_1c_ level is a recognized indicator of glycemic control over a retrospective period, reflecting average blood glucose levels over approximately 3 months, it is regarded as a standard for assessing the effectiveness of long-term diabetes interventions [77]. This measure offers a more stable view of a patient’s glycemia levels than instantaneous measurements, which can be influenced by many immediate factors [77]. Longer interventions could allow for more accurate adjustments in treatments and disease management behaviors as well as provide enough time for these changes to result in improvements in glycemic control.

The health professionals involved in these studies were primarily physicians, dietitians, and nurses. Nearly half (19/42, 45%) of the studies involved a multidisciplinary care team [29,31,34,37,38,41,44,48,50,52-54,57-59,63,67,68,71] (Multimedia Appendix 4). The studies showed that health technologies could help optimize the therapeutic education and monitoring of people living with type 2 diabetes through collecting and sharing information between consultations. Care provider personnel would thus be better able to focus on other aspects of their practice during consultations. Some of the interventions (4/42, 10%) used a videoconferencing platform for consultations with the health professional to make the exchanges more natural and pleasant [55,60,67,68]. A recent narrative review that included 12 randomized controlled trials assessing the effectiveness of telemedicine versus conventional counseling, demonstrated that the counseling and monitoring of patients living with diabetes via telemedicine was more effective than conventional counseling [78]. Similarly, health technologies could help improve the efficiency of practical tasks performed by health professionals, for example, by producing more concise automatically generated reports that can be shared among the care team, thus fostering interdisciplinary monitoring and follow-up. They also offer the possibility of monitoring patients in real time and sharing targeted information with them, thereby facilitating timely adjustments. Telehealth tools enable the continuous monitoring of blood glucose levels, physical activity, diet, medication intake, and other health indicators. This enables patients and health care providers to quickly detect fluctuations in blood sugar levels and take appropriate action to maintain optimal control of blood sugar levels [1]. The features of telehealth tools can provide personalized recommendations and advice based on each patient’s specific data [2]; for example, patients can receive medication reminders, nutritional advice tailored to their dietary preferences, and suggestions for physical activities based on their condition and health goals [2]. Telehealth tools offer educational resources and information on type 2 diabetes [3]. Patients can access educational materials, explanatory videos, meal plans, and tips to improve their understanding of the disease and its management [3]. This promotes patient empowerment by enabling them to actively participate in the management of their health [3-5]. Telehealth tools can include features such as appointment reminders, food diaries, and physical activity logs. These features help patients track their progress, stay engaged with their treatment, and maintain their motivation [3,5].

Our findings are in line with the chronic care model [79]. Telehealth interventions, as observed in our study, frequently incorporate goal-setting tools that empower patients to set and track health-related objectives, aligning with the model’s emphasis on self-management support. In addition, our results underscore the vital role of health professional support within telehealth interventions, enabling remote monitoring and timely guidance, consistent with the model’s focus on patient-centered care. Social support emerged in our findings, with patients benefiting from the encouragement of their social networks—a concept aligned with the chronic care model’s recognition of involving the patient’s social support system. Finally, our research highlights the inclusion of educational materials in telehealth interventions, providing patients with essential knowledge about their condition, in line with the model’s emphasis on patient education. Together, these elements within telehealth strategies contribute to patient empowerment, improved self-management, and enhanced outcomes for the management of chronic conditions such as diabetes, emphasizing the importance of a comprehensive approach to health care delivery, even in remote or web-based settings.

However, there are also potential limitations and challenges associated with the use of telehealth tools for the management of type 2 diabetes. The use of telehealth tools may be limited by internet access, technological skills, and the availability of the necessary devices [2,3]. Populations that have been historically marginalized or disadvantaged may face digital disparities, limiting their ability to benefit fully from these tools. It is thus essential to recognize that some patients may require additional human support. Interaction with health care providers may be necessary to obtain answers to questions, resolve problems, and receive emotional support. Furthermore, the use of telehealth tools involves the collection, storage, and sharing of sensitive health data. It is crucial to implement robust security measures to protect data confidentiality and prevent privacy breaches [2,8]. Telehealth tools use monitoring devices to collect data, such as blood glucose meters or continuous blood glucose monitoring sensors. However, these devices can have technical limitations and measurement errors, which can affect the accuracy of the data collected and potentially influence treatment decisions [5,8]. Given that diabetes management is characterized by a long process of therapeutic education, monitoring, and follow-up, technological support would be a helpful asset in primary health care because it would help maintain motivation [29,37,40,46,54,61,70] through the use of numerous tools (goal-setting tools and shared decision-making support tools, recipes, informational content, etc), by facilitating interactions with a health professional, and by promoting access to care (eg, with the possibility of using multilingual resources).

### Recommendations for Future Designs

Telehealth offers many opportunities for diabetes self-management and monitoring, enabling patients to benefit from remote care, continuous monitoring, and personalized support. The use of continuous blood glucose monitoring devices, mobile apps, web-based platforms, and other technologies facilitates the collection and tracking of diabetes-related data [9]. The introduction of web-based educational resources, web-based learning modules, and self-help tools to help patients better understand their disease as well as manage their diet, physical activity, medication, and monitoring of blood glucose levels promotes patient self-management and empowerment [10,11]. In addition, web-based support via secure messaging to answer patients’ questions and respond to their concerns supports therapeutic education and keeps them engaged. Indeed, technology developers will need to set up clear and effective communication channels between patients and health professionals. This may include web-based consultations, secure message exchanges, and regular reports on patient progress [11]. Finally, it will be important to consider the integration of these telehealth interventions into existing health care systems, ensuring coordination and continuity of care. It will be necessary to ensure that data collected by remote monitoring devices are accessible to health professionals and integrated into patients’ medical records [12].

### Limitations of Included Studies

The studies identified in this review involved voluntary patient participation. In particular, the studies favored individuals with good technology literacy. The selection bias inherent in voluntary patient participation and the preference for technology-literate individuals suggest that the findings might not be generalizable to the broader population of people with diabetes. The indicators used to assess the effectiveness of the interventions were primarily dietary intake; clinical indicators such as glycemia levels, HbA_1c_ levels, blood pressure, and cholesterol levels; physical activity; medication adherence; motivation; and the use of telehealth technology. Although positive changes in these indicators were noted in most clinical results, this may translate into something other than rigorous clinical parameters. Different strategies were used to collect data, notably involving innovative digital tools (although these tools did not undergo a validation study). In addition, lifestyle changes (dietary planning and physical activity) were measured using the patient self-administered digital questionnaires, leaving the door open to all biases inherent in self-reporting. A meta-analysis of these data would help inform a position in this regard.

The heterogeneity of the included studies posed a real challenge in interpreting the results. Aside from the various methods used, which yielded different levels of evidence, the interventions were based mainly on effecting behavior changes through therapeutic education supported by digital tools and a health professional; yet, none of the studies assessed the impact on the results of the context within which these technologies were used, such as concurrent public health policies (eg, diabetes or obesity prevention campaigns, the promotion of a balanced diet, physical activity, or tobacco use).

Moreover, the literature states that 90% of people with diabetes have at least 1 other chronic disease. Nonetheless, few interventions have provided the integrated management of diabetes and other pathologies. Specifically, renal and cardiac risks have not always been assessed. The multipathological context should be systematically considered when designing studies because multiple medication use (eg, sulfonylureas and insulin) can cause iatrogenic hypoglycemia and influence the clinical parameters [80-82]. Similarly, the different stages of diabetes severity should be documented to foster a more accurate interpretation of the results.

The varying durations of the interventions, ranging from 1 month to 18 months, and the differing technologies used emphasize that outcomes such as improvements in HbA_1c_ levels are not uniform across all studies. The positive association observed with longer interventions and the reduction in HbA_1c_ levels may not hold true in every context or for every patient demographic. The role of health professionals in these interventions is undoubtedly significant, but the translation of these findings into practice must consider the individual needs and circumstances of diverse patient populations, including access issues and technological literacy. The integration of everyday technologies seems promising for broader implementation; however, this assumption requires careful consideration of the digital disparities that may exist, particularly among groups that have been historically marginalized or disadvantaged.

### Strengths and Limitations of This Review

To further leverage the qualitative nature of the content analyzed in the studies, we performed a descriptive content analysis of the data using NVivo (release 1.7). This allowed us to supplement our research with a narrative account of the selected studies. The abundance of literature on the subject attests to a worldwide questioning of digital health policies. The COVID-19 pandemic led to a doubling of the number of annual publications on the topic of telehealth interventions used for type 2 diabetes or prediabetes self-management and monitoring with health professional involvement. Given the rapid development of technologies and research, which has only escalated in recent years, a systematic review would help provide invaluable data on the effectiveness of these interventions. This scoping review included studies published in peer-reviewed journals and is thus subject to publication bias owing to the well-documented notion that researchers and journals tend to publish positive results. In addition, we limited ourselves to selecting studies published in French or English from 2010 given the rapid pace of technological development and the consequent rapid increase in the literature. Future researchers should consider more inclusive approaches, such as conducting systematic reviews that encompass gray literature and unpublished studies. This ensures a more comprehensive and unbiased overview of existing literature on the topic.

The results of this review did not allow us to identify how the 8 key digital tool features should be developed and combined to help improve health outcomes. However, the strategy most often combined with telehealth interventions facilitating interaction with health professionals was the monitoring of glycemia levels, diet, and physical activity. A few of the studies (7/42, 17%) also included medication monitoring and therapeutic education. Future studies should perform in-depth analyses of the usability and acceptability of these technologies to highlight the design issues and shed light on health policies.

The diversity of the interventions analyzed underscores the necessity to acknowledge the unique challenges and issues inherent to each specific population. Such issues can encompass socioeconomic factors, cultural differences, accessibility to health services, and varying levels of health literacy, all of which can significantly influence the effectiveness of interventions; for instance, interventions that succeed in urban environments with high connectivity and technologically savvy populations may not yield identical results in rural or low-income areas where internet access is scarce and digital literacy is an issue. Moreover, the cultural context may impact patient engagement and the suitability of educational materials. Each population may hold distinct health beliefs, practices, and priorities, which must be considered during the design and implementation of health interventions. Recognizing these disparities is critical to understanding why results from 1 group cannot be generalized to another. Public health strategies must develop resource allocation policies and create interventions focused on the users’ needs. Hence, although telehealth presents a promising avenue for improving diabetes management, its application must be nuanced and considerate of the public health challenges unique to each specific population to be truly effective and equitable.

### Future Research Prospects

With regard to gaps in the literature, some questions require further research. This scoping review revealed a need for long-term implementation studies, possibly because telehealth programs require a less-structured time commitment and could be used over extended periods. Long-term evaluation studies are also needed to facilitate the implementation of telehealth interventions. Further studies on adherence and engagement could explore the factors that influence patients’ adherence to telehealth interventions and their engagement in diabetes self-management. These studies will also help to identify effective strategies for encouraging patients’ active participation and maintaining their motivation over the long term. Evaluation frameworks should incorporate reports on participant engagement and satisfaction, acceptability, security, and costs into future telehealth interventions because these will facilitate their translation into clinical practice. In addition, the measurement of the effects of interventions should include measures other than clinical data, such as patient-reported experience measures and patient-reported outcome measures to ensure that these interventions are meeting the needs of patients. In addition, multimorbidity was mentioned by only a few of the included studies (7/42, 17%) and warrants further research to assess the impact of these interventions on health [34,49,54,56,65,67,70]. Additional studies could define standardized assessment criteria for telehealth interventions that support the therapeutic management of patients with diabetes and multiple comorbidities. The impact of equity of access to care on the use of telehealth interventions for populations considered vulnerable, including populations with low-income status, rural or remote populations, and culturally diverse groups, will need to be studied. A better understanding of these impacts will help identify potential barriers and strategies to reduce disparities and improve equitable access to telehealth [12]. Finally, it will be vital to evaluate the effectiveness of integrating telehealth interventions into existing health care systems, including collaboration among health professionals, data sharing, and care coordination. This will help distinguish best practices for the successful integration of telehealth into clinical care and existing health care systems [12]. Of the 42 studies, 3 (7%) assessed the impact on the cost of care [48,58,64]. The macroeconomic implications of these telehealth interventions for health care systems warrant future studies to shed clearer light on health policies. Finally, the COVID-19 pandemic has revealed the various structural and organizational shortcomings of health care around the globe. It has also accelerated the dissemination and adoption of digital tools and advanced the digital ambitions of governments worldwide. The abundance of publications means that future studies can perform a meta-analysis of randomized controlled trials. Our analysis underscores the critical role of multidisciplinary health care teams and promotes the integration of ubiquitous technologies into daily health management practices to achieve superior patient outcomes. Furthermore, this review stresses the necessity of considering the long-term viability of telehealth solutions, patient adherence, and the seamless incorporation of these solutions into current health care frameworks in subsequent research.

Finally, although we included studies conducted in different parts of the world in this scoping review, we did not find relevant studies conducted in Canada, indicating an opportunity for research tailored to the Canadian context. For the implementation of future telehealth interventions to improve diabetes management in Canada, it is recommended to consider the specificities of the Canadian health care system, such as the heterogeneity of its organization across different provinces, the diversity of its population, and its varied health resources. It would be wise to design personalized interventions that address the unique needs of patients with diabetes within the Canadian population, particularly in Indigenous communities that are disproportionately affected by diabetes, including linguistic and cultural considerations. Strategies for equitable access to telehealth technologies for populations that have been historically marginalized or those living in remote areas should also be considered. Training health professionals in telehealth tools and best practices for web-based care is equally essential. Moreover, interdisciplinary and intersectoral collaboration would be beneficial to effectively integrate telehealth into primary care, allowing for coordinated and consistent follow-up. Finally, by anticipating challenges related to privacy and data security, interventions should incorporate robust security measures to protect sensitive patient information while focusing on a personalized approach and the development of patient-centered interventions and technologies.

### Conclusions

This review systematically maps out the effectiveness of telehealth interventions for managing type 2 diabetes, with a focus on the enhanced outcomes gained through the involvement of health professionals. It presents a detailed categorization of the pivotal characteristics of digital tools into 8 distinct areas that significantly influence the success of these interventions. The evidence-based data suggest that participation in sustained telehealth interventions with health professional involvement helps improve health outcomes and type 2 diabetes–related behavior, reducing the risks of complications. However, despite our identification of the key digital tool features of these interventions, it remains to be seen how to combine and translate them into long-term usable components in specific care contexts. Nonetheless, the results are promising for future health care because they point to consolidating care through a single platform, which could improve patients’ quality of life while encouraging active self-management. They also shed light on developing evidence-based telehealth programs that can be adapted to specific care contexts and offer decision makers more effective options for funding diabetes management programs. Ultimately, this review aims to enrich the understanding of telehealth’s role in diabetes care and to outline specific domains for future research that will inform policy making and the advancement of telehealth practices.

## Appendix

Multimedia Appendix 1 PRISMA-ScR (Preferred Reporting Items for Systematic Reviews and Meta-Analyses Extension for Scoping Reviews) checklist.

Multimedia Appendix 2 Search strategy.

Multimedia Appendix 3 Data extraction table.

Multimedia Appendix 4 Health professional involvement.

Multimedia Appendix 5 Digital features of the interventions.

Multimedia Appendix 6 Studies showing significant positive health outcomes.

## Abbreviations

DASH: Dietary Approaches to Stop Hypertension
HbA_1c_: glycated hemoglobin
PRISMA-ScR: Preferred Reporting Items for Systematic Reviews and Meta-Analyses Extension for Scoping Reviews
