# Supplementary material for: The Key Digital Tool Features of Complex Telehealth Interventions Used for Type 2 Diabetes Self-Management and Monitoring With Health Professional Involvement: Scoping Review
Source: JMIR Med Inform. 2024 Mar 13;12:e46699. doi: 10.2196/46699 (PMC10973964; doi:10.2196/46699)
Supplement: Multimedia Appendix 2 [file medinform_v12i1e46699_app2.docx]

**Multimedia Appendix 2 - Search Strategy**

| 1. | exp Cell Phone/ |
| --- | --- |
| 2. | exp Smartphone/ |
| 3. | exp Computers/ or exp Computers, Handheld/ |
| 4. | exp Mobile Applications/ |
| 5. | exp Telemedicine/ |
| 6. | exp Telerehabilitation/ |
| 7. | exp Remote Consultation/ |
| 8. | exp Videoconferencing/ |
| 9. | exp Internet-Based Intervention/ |
| 10. | exp Video Recording/ |
| 11. | ("cell phone*" or cellphone* or "mobile phone*" or "smart phone*" or smartphone* or "mobile app*" or "mobile device*" or "online portal*" or "web* portal*" or chatbot* or robot* orsmartwatch* or "smart watch*" or ereferr* or "e-referr*" or chat or avatar* or "online platform*" or "mobile platform*" or telehealth* or "tele-health*" or teletherapy or "tele-therapy"or telemedicine or "tele-medicine" or telerehabilitation or "tele-rehabilitation" or teletherap* or "tele-therap*" or telenutrition* or "tele-nutrition*" or telediet* or "tele-diet*" or ehealth*or "e-health*" or mhealth* or "m-health*" or "digital health*" or "digital rehabilitation" or "online health*" or "web health*" or "mobile health*" or "virtual care" or videoconferenc* or"video conferenc*" or videoconsult* or "video consult*" or teleconsultation* or "tele consultation*" or econsultation* or "e-consultation*" or telecare or "tele care" or erehabilitationor "e-rehabilitation" or teleintervention* or "tele intervention*" or teletreatment* or "tele treatment*" or "internet intervention*" or "internet-based intervention*" or "internetconsultation*" or "internet-based consultation*" or "web* intervention*" or "web*-based intervention*" or "web* consultation*" or "web*-based consultation*" or "onlineintervention*" or "online-based intervention*" or "online consultation*" or "online-based consultation*" or "computer* intervention*" or "computer*-based intervention*" or"computer* consultation*" or "computer*-based consultation*" or "cyber* intervention*" or "cyber*-based intervention*" or "cyber* consultation*" or "cyber*-based consultation*"or "virtual* intervention*" or "virtual*-based intervention*" or "virtual* consultation*" or "virtual*-based consultation*" or "digital* intervention*" or "digital*-based intervention*" or"digital* consultation*" or "digital-based consultation*" or "remote* intervention*" or "remote*-based intervention*" or "remote* consultation*" or "remote*-basedconsultation*").ab,kw,ti. |
| 12. | 1 or 2 or 3 or 4 or 5 or 6 or 7 or 8 or 9 or 10 or 11 |
| 13. | exp Diabetes Mellitus/ |
| 14. | (diabetic* or diabetes).ab,kw,ti. |
| 15. | 13 or 14 |
| 16. | 12 and 15 |
| 17. | exp Nutrition Assessment/ or exp Nutrition Therapy/ |
| 18. | exp Nutritionists/ |
| 19. | exp Feeding Behavior/ or exp Diet/ or exp Energy Intake/ |
| 20. | exp Dietetics/ |
| 21. | (nutrition* or diet* or carb* intake or eating).ab,kw,ti. |
| 22. | 17 or 18 or 19 or 20 or 21 |
| 23. | 12 and 15 and 22 |
| 24. | limit 23 to (english or french) |
